# A Novel PEGylation Method for Improving the Pharmacokinetic Properties of Anti-Interleukin-17A RNA Aptamers

**DOI:** 10.1089/nat.2016.0627

**Published:** 2017-02-01

**Authors:** Kazuhiko Haruta, Natsuki Otaki, Masakazu Nagamine, Tomoyoshi Kayo, Asako Sasaki, Shinsuke Hiramoto, Masayuki Takahashi, Kuniyoshi Hota, Hideaki Sato, Hiroaki Yamazaki

**Affiliations:** ^1^ R&D Center, Zenyaku Kogyo Co., Ltd., Tokyo, Japan.; ^2^ GeneDesign, Inc., Osaka, Japan.; ^3^ Prescription Products Development, Zenyaku Kogyo Co., Ltd., Tokyo, Japan.

**Keywords:** aptamer, interleukin-17A, PEGylation, pharmacokinetics

## Abstract

The obstacles to the development of therapeutic aptamers for systemic inflammatory diseases, such as nuclease degradation and renal clearance, have not been fully overcome. Here, we report a novel PEGylation method, sbC-PEGylation, which improves the pharmacokinetic properties of RNA aptamers that act against interleukin-17A (IL-17A) in mice and monkeys. sbC-PEGylated aptamers were synthesized by coupling the symmetrical branching molecule 2-cyanoethyl-*N*,*N*-diisopropyl phosphoroamidite to the 5′ end of the aptamer, before conjugating two polyethylene glycol (PEG) molecules to the aptamer. Pharmacokinetic studies showed that compared with conventionally PEGylated aptamers, the sbC-PEGylated aptamer exhibited excellent stability in the blood circulation of mice and monkeys. In addition, one of the sbC-PEGylated aptamers, 17M-382, inhibited the interleukin-6 (IL-6) production induced by IL-17A in NIH3T3 cells in a concentration-dependent manner, and the half-maximal inhibitory concentration of sbC-PEGylated 17M-382 was two times lower than that of non-PEGylated 17M-382. Furthermore, the intraperitoneal administration of sbC-PEGylated 17M-382 significantly inhibited the IL-6 production induced by IL-17A in a mouse air pouch model. Our findings suggest that the novel PEGylation method described in this study, sbC-PEGylation, could be used to develop anti-IL-17A aptamers as a therapeutic option for systemic inflammatory disease.

## Introduction

Aptamers are artificial oligonucleotides that specifically bind to target molecules with a high affinity. Consequently, they can inhibit the binding of a protein to its receptor, similar to therapeutic neutralizing antibodies. However, aptamers are considered to have some clinical advantages over therapeutic antibodies, for example, they exhibit low immunogenicity [1–3].

A large variety of therapeutic aptamers have been reported since the systematic evolution of ligands by exponential enrichment (SELEX), a technique used to identify aptamers, was established [4,5]. However, only pegaptanib, an anti-vascular endothelial growth factor (VEGF) aptamer, has been approved for the treatment of age-related macular degeneration [6]. In addition, recent developments in this area have focused on treatments for ophthalmic disease [7]. These points indicate that the obstacles to the development of therapeutic aptamers for other diseases, especially systemic diseases, such as nuclease degradation and renal clearance, have not been fully overcome.

PEGylation, in which polyethylene glycol (PEG) is conjugated to a pharmaceutical, is one of the strategies used to inhibit the enzyme degradation and renal clearance of drugs and, hence, to improve their stability *in vivo.* In studies of protein-based therapeutics, it was reported that the molecular weight (MW) and configuration of PEG affected not only the pharmacokinetic (PK) properties of the drug but also its biological activity. Thus, it is important to select PEG with appropriate MW and configurations for the desired clinical benefits [8–11]. On the other hand, there is limited information about the PEGylation of aptamers, although various techniques involving oligonucleotide modification have been developed to protect aptamers from nuclease degradation [12–16]. In this study, to optimize the *in vivo* PK properties of aptamers, we developed a novel PEGylation method, **s**ymmetrical **b**ranching 2-cyanoethyl-*N*,*N*-diisopropyl (**C**ED) phosphoramidite PEGylation (sbC-PEGylation), and we evaluated the effects of sbC-PEGylation on the PK properties of RNA aptamers that inhibit interleukin-17A (IL-17A), which were originally reported by Ishiguro *et al.* [17]. It was found that sbC-PEGylation markedly improved the PK properties and enhanced the neutralizing activity of anti-IL-17A aptamers. These results suggest that sbC-PEGylation might represent a breakthrough in the development of therapeutic aptamers.

## Materials and Methods

### PEGylation and oligonucleotides

**FIG. 1.**
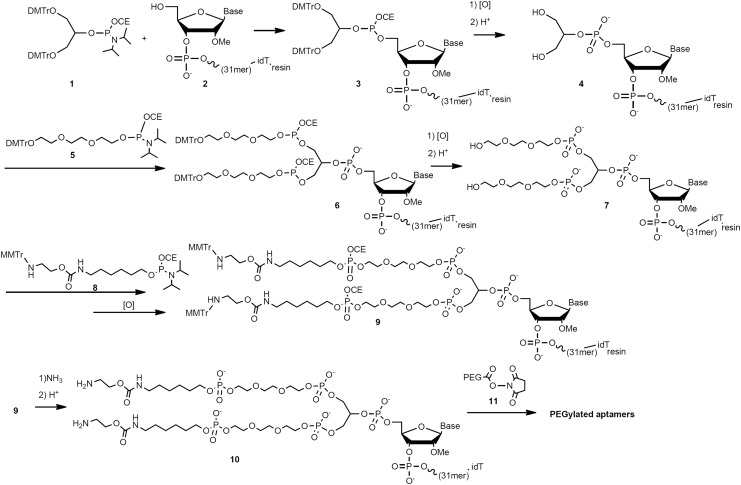
Scheme for sbC-PEGylation. DMTr, 4,4′-dimethoxytrityl; MMTr, *p*-methoxyphenyl diphenylmethyl; OCE, cyanoethoxy.

The PEGylation and synthesis of the oligonucleotides were performed by GeneDesign, Inc. (Osaka, Japan). The PEG molecules used in this study were obtained from NOF Corporation (Tokyo, Japan) or JenKem Technology (Beijing, China). Symmetrical branching CED phosphoramidite, DMT-triethyloxy-glycol phosphoramidite (ChemGenes Corporation, Wilmington, MT), and ssH linker phosphoramidite (Sigma-Aldrich Corporation, St. Louis, MO) were used for the sbC-PEGylation of the anti-IL-17A aptamers, according to the scheme described in Fig. 1. In brief, the 5′-OH oligonucleotide (compound **2**) was sequentially synthesized from a 3′-inverted deoxythymidine (idT)-loaded controlled pore glass that served as a starting material by using an automated solid-phase oligonucleotide synthesizer, before being subjected to acid detritylation. In the same synthesizer, symmetrical branching CED phosphoramidite (compound **1**, 6 equivalents) was reacted with compound **2** to give compound **3,** which had a 5′-O-branched oligonucleotide. After oxidation and deprotection of compound **3**, the resultant compound **4** was reacted with DMT-triethyloxy-glycol phosphoramidite (compound **5**, 9 equivalents), giving compound **6**. Oxidation and deprotection of compound **6** proceeded in the same manner as described earlier, and the resultant compound **7** was reacted with ssH linker phosphoramidite (compound **8**, 9 equivalents) and oxidized successively, giving compound **9**. Then, compound **9** was cleaved from the resin and base deprotected by using ammonium hydroxide. Next the 2′-tert-butyldimethylsilyl (TBDMS) group was deprotected with acid, and then the product was purified by using reverse-phase column chromatography to give the purified amine compound **10**, which was used as a PEGylation precursor. Next, an aqueous solution of compound **10** and a dimethyl sulfoxide-acetonitrile (4:1) solution of activated PEG (compound **11**, 9 equivalent) were added to 0.1 M sodium carbonate buffer solution (pH 9.0), and the mixture was stirred at 25°C for 2 h to give the crude PEGylated product. After the crude PEGylated product had been purified with reverse-phase column chromatography, the desired purified PEGylated aptamers were obtained. In some studies, compound **2** was conventionally PEGylated by using ssH linker phosphoramidite alone. The weights of the sbC-PEGylated and PEGylated aptamers were calculated as oligonucleotide weight values.

The RNA sequence of the anti-IL-17A aptamers (17M-200-S1 and 17M-382) used in this study, 5′-GGGUAGCCGGAGGAGTCAGTAAUCGGUACCC-3′, was based on the Apt21-2 sequence reported by Ishiguro *et al.* [17]. To prevent nuclease degradation, fluorine, methoxy, or hydrogen groups were introduced at the 2′-OH positions of some riboses, and the aptamers' phosphodiester backbones were partially replaced with phosphorothioate.

### PK studies in mice

The PK studies were performed according to the procedures approved by the Zenyaku Kogyo Co., Ltd., animal care and use committee. Male C57BL/6J mice (6 weeks old) were purchased from Charles River Laboratories, Japan (Kanagawa, Japan). They were maintained at around 22°C under a 12 h light/dark cycle and given standard chow and tap water *ad libitum*. sbC-PEGylated or PEGylated aptamers were dissolved in saline at a concentration of 0.2 mg/mL. They were intravenously administered to the mice at a dose of 1 mg/kg. Blood was collected from the mice at the indicated time points (*n* = 3 per time point), and ethylenediaminetetraacetic acid (EDTA) plasma samples were prepared and stored at −70°C until the assays.

### PK studies in monkeys

The PK studies in cynomolgus monkeys were conducted at Ina Research, Inc. (Nagano, Japan), according to the procedures approved by the Ina Research, Inc., institutional animal care and use committee. Male cynomolgus monkeys (age: 3–5 years; body weight: 2.6–4.4 kg) were used in this study. sbC-PEGylated or conventionally PEGylated 17M-382 was dissolved in saline at a concentration of 1 mg/mL. The aptamers were intravenously administered to the monkeys (*n* = 3) at a dose of 1 mg/kg. Two weeks after their intravenous administration, they were also subcutaneously administered at a dose of 1 mg/kg. Blood was collected at the indicated time points, and EDTA plasma samples were prepared and stored at −70°C until the assays.

### Enzyme-linked oligo-sorbent assay

The plasma levels of aptamers were measured by using an enzyme-linked oligo-sorbent assay (ELOSA) according to the method reported by Healy *et al.* [18]. The RNA sequences of the detection probe and capture probe were 5′-6-FAM-spacer 18-GGGUACCGAUUACUG-3′ and 5′-ACUCCUCCGGCUACCC-spacer 9-amino C6 linker-3′, respectively. The PK parameters were derived by using noncompartmental models with WinNonlin^®^ version 6.4 on Phoenix^®^ 1.4 (Pharsight Corp., Mountain View, CA).

### *In vitro* IL-17A-neutralizing activity

The effects of sbC-PEGylation on the IL-6 production induced by human IL-17A in NIH3T3 cells were examined. Namely, 10 ng/mL of human IL-17A (R&D Systems, Inc., Minneapolis, MN) was incubated with various concentrations of the aptamers for 30 min at 37°C. Then, suspensions of NIH3T3 cells (American Type Culture Collection, Manassas, VA) (density: 5 × 10^5^ cells/mL) and mouse tumor necrosis factor (TNF)-α (2 ng/mL; R&D Systems, Inc.) were added to the plates. After 24 h, the supernatants were collected and stored at −70°C until the assays were performed. Then, the levels of mouse IL-6 in the supernatants were measured by using an ELISA kit (Invitrogen Corporation, Frederick, MD).

### *In vivo* IL-17A-neutralizing activity

According to the method reported by Maione *et al.* [19], we examined the effects of sbC-PEGylation on the IL-17A-neutralizing activity of 17M-382 by using a mouse air pouch model. sbC-PEGylated 17M-382 was intraperitoneally administered at 1, 24, 72, or 168 h before the administration of human IL-17A (500 μg/pouch, kindly donated by Dr. Ishiguro, Tokyo University) into each air pouch. Then, the exudates from each air pouch were collected at 24 h after administration of human IL-17A and they were stored at −70°C until the concentration of IL-6 was measured by using an ELISA kit (Invitrogen Corporation).

### Statistical analysis

Data are expressed as the mean ± standard deviation (SD, *n* = 3, for the PK studies and *in vitro* studies) or the mean ± standard error of the mean (SEM, *n* = 7 or 8, for the mouse air pouch model). The statistical significance of differences was assessed by using one-way analysis of variance (ANOVA) followed by Dunnett's test. These statistical analyses and the determination of half-maximal inhibitory concentration (IC_50_) values (using the four-parameter curve fit algorithm) were performed by using the Kaleida Graph 3.6 software (Synergy Software, Reading, PA). *P*-values of <0.05 were considered significant.

## Results

### sbC-PEGylation

**FIG. 2.**
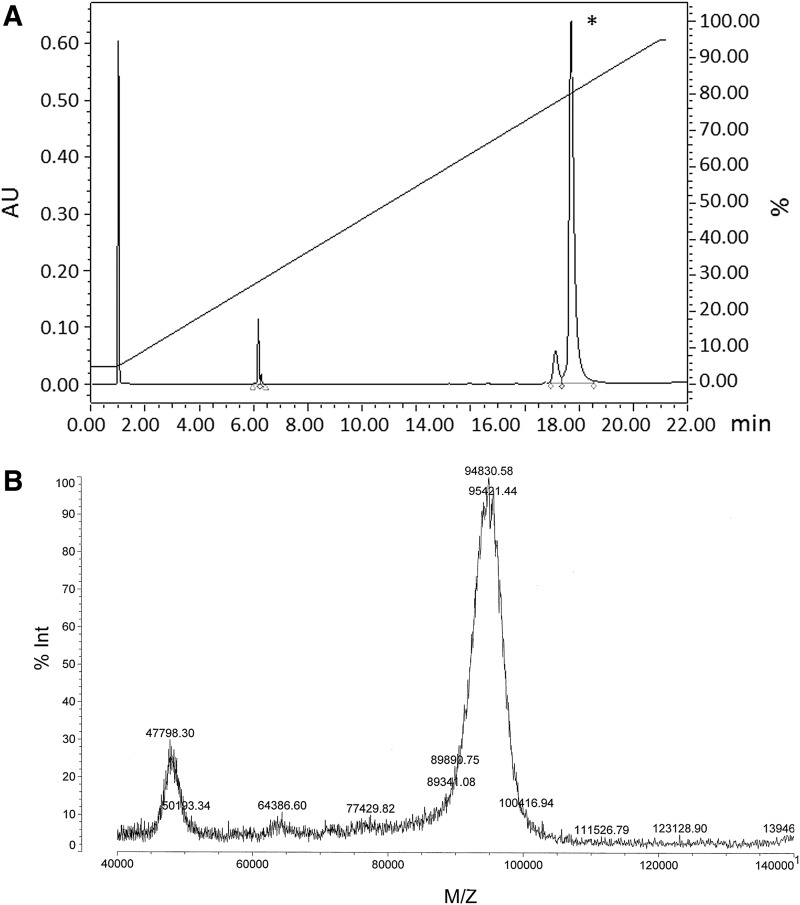
**(A)** Reverse-phase HPLC and **(B)** matrix-assisted laser desorption/ionization time-of-flight mass spectrometry (MALDI-TOF-MS) analyses of sbC-PEGylated 17M-382. *The yield of sbC-PEGylated 17M-382 was 87.4%.

Figure 2A shows the results of the reverse-phase high performance liquid chromatography (HPLC) analysis of 17M-382 after it had been sbC-PEGylated with two-armed 40-kDa PEG (Y-shaped PEG; JenKem Technology). The yields of sbC-PEGylated 17M-200-S1 and sbC-PEGylated 17M-382 were 85.0% and 87.4%, respectively. After purifying them with reverse-phase HPLC, we confirmed that the sbC-PEGylated aptamers contained two 40-kDa PEG molecules using matrix-assisted laser desorption/ionization time-of-flight mass spectrometry (MALDI-TOF-MS, Fig. 2B).

### Effects of sbC-PEGylation on the PK properties of aptamers in mice

**FIG. 3.**
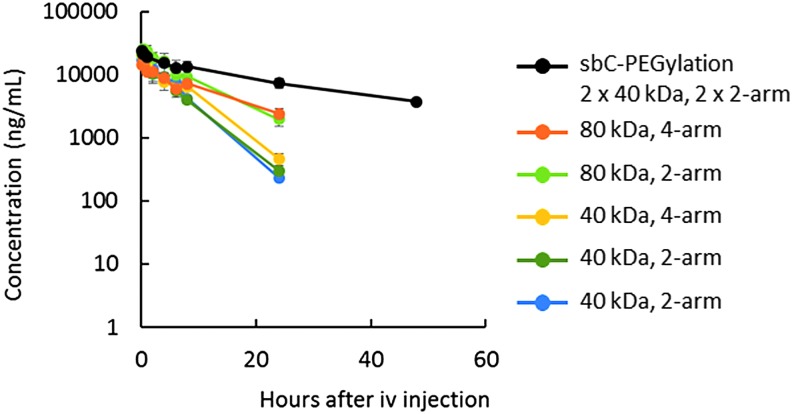
Plasma concentration-time curves of sbC-PEGylated and conventionally PEGylated aptamers after their intravenous injection into mice. 17M-200-S1 that had been sbC-PEGylated with two 2-armed 40-kDa PEG (Y-shaped PEG, block) or conventionally PEGylated with various PEG (*orange*: SUNBRIGHT^®^ GL4-800GS2; *yellow*-*green*: SUNBRIGHT GL2-800GS2; *yellow*: SUNBRIGHT GL4-400GS2; *green*: SUNBRIGHT GL2-400GS2; *blue*: Y-shaped PEG) was intravenously administered to mice at a dose of 1 mg/kg. Three mice were used at each time point, and the data are expressed as mean ± SD values (*n* = 3).

To evaluate the effects of sbC-PEGylation on the renal clearance and nuclease degradation of aptamers *in vivo*, we first used 17M-200-S1, which was easily degraded in mouse serum (Supplementary Fig. S1; Supplementary Data are available online at www.liebertpub.com/nat). sbC-PEGylated 17M-200-S1 had the longest plasma half-life (t_1/2_, 22.5 h) of the aptamers that were conjugated with 80-kDa PEG. The plasma t_1/2_ of sbC-PEGylated 17M-200-S1 was approximately two to three times longer than those of the two-armed 80-kDa PEG (7.5 h) and the four-armed 80-kDa PEG (10.5 h). Compared with the aptamers that were PEGylated with 40-kDa PEG, the sbC-PEGylated 17M-200-S1 remained in circulation for 4.5 to 6.0 times longer (Fig. 3 and Table 1).

**Table 1.** Plasma Half-Lives (t_1/2_) of sbC-Pegylated and Pegylated Aptamers in Mice

	*PEG*			
*Aptamers*	*Product name*	*MW (kDa)*	*No. of arms*	*t_1/2_ (h)*
sbC-PEGylated 17M-200-S1	Y-shaped PEG+Y-shaped PEG	80 (40 + 40)	4 (2 + 2)	22.5
PEGylated 17M-200-S1	SUNBRIGHT^®^ GL4-800GS2	80	4	10.5
	SUNBRIGHT GL2-800GS2	80	2	7.5
	SUNBRIGHT GL4-400GS2	40	4	5.0
	SUNBRIGHT GL2-400GS2	40	2	4.3
	Y-shaped PEG	40	2	3.7
sbC-PEGylated 17M-382	Y-shaped PEG+Y-shaped PEG	80 (40 + 40)	4 (2 + 2)	22.8
	SUNBRIGHT ME-400GS+SUNBRIGHT ME-400GS	80 (40 + 40)	2 (linear+linear)	4.9
PEGylated 17M-382	SUNBRIGHT GL4-800GS2	80	4	20.9

SUNBRIGHT^®^ is a registered trademark of the NOF Corporation.

MW, molecular weight.

Next, we used 17M-382, which was not degraded in mouse serum for 72 h (Supplementary Fig. S1), to evaluate the effects of sbC-PEGylation on renal clearance. The plasma t_1/2_ of sbC-PEGylated 17M-382 (22.8 h) and sbC-PEGylated 17M-200-S1 (22.5 h) did not differ virtually. In addition, the plasma t_1/2_ of sbC-PEGylated 17M-382 was almost as long as that of 17M-382 PEGylated with four-armed 80-kDa PEG (20.9 h, Table 1). However, the use of linear 40-kDa PEG instead of two-armed PEG markedly reduced the plasma t_1/2_ of sbC-PEGylated 17M-382 (4.9 h). Taken together, it was considered that sbC-PEGylation with two 2-armed 40-kDa PEG molecules increased the aptamers' MW to a greater extent (total: 80 kDa) and induced more marked steric hindrance (caused by the 4 arms around the aptamers) than conventional PEGylation, resulting in reductions in renal clearance and nuclease degradation.

### Effects of sbC-PEGylation on the PK properties of aptamers in monkeys

**FIG. 4.**
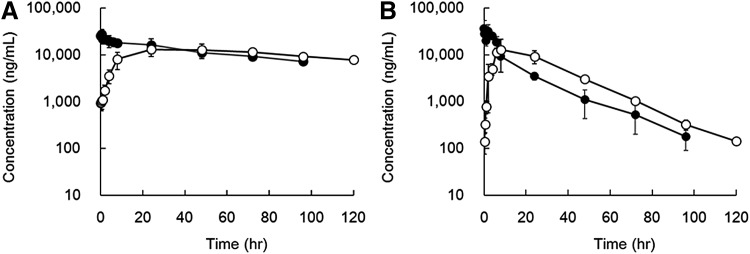
Plasma concentration-time curves of sbC-PEGylated and conventionally PEGylated aptamers after their injection into monkeys. 17M-382 that had been sbC-PEGylated with two 2-armed 40-kDa PEG **(A)** or conventionally PEGylated with a two-armed 40-kDa PEG **(B**, SUNBRIGHT GL2-400GS2**)** was intravenously (*closed circles*) or subcutaneously (*open circles*) administered to monkeys at a dose of 1 mg/kg. Data are expressed as mean ± SD values (*n* = 3).

Since it was predicted that the most suitable oligonucleotide/PEGylation combination would optimize the aptamers' PK properties in monkeys, we used 17M-382, and we compared the PK properties of sbC-PEGylated aptamers with those of conventionally PEGylated aptamers. When sbC-PEGylated 17M-382 was administered intravenously and subcutaneously, it exhibited plasma t_1/2_ of 72.0 ± 10.3 and 107 ± 52.8 h, respectively, which were 4.1 and 6.5 times longer than those obtained for 17M-382 PEGylated with two-armed 40 kDa PEG, respectively. Similarly, the area under the curve values of sbC-PEGylated 17M-382 were 5.3 to 5.5 times longer than those of PEGylated 17M-382. On the contrary, the CL/F value of sbC-PEGylated 17M-382 was approximately one-fifth lower than that of PEGylated 17M-382. When the aptamers were administered subcutaneously, the T_max_ of sbC-PEGylated 17M-382 was 3.8 times longer than that of PEGylated 17M-382. However, there were no differences in C_max_, Vz/F, or Vss (intravenous administration) between the aptamers (Fig. 4 and Table 2).

**Table 2.** Pharmacokinetic Properties Of sbC-Pegylated And Pegylated Aptamers In Monkeys

	*PEG*										
*Aptamers*	*Product name*	*MW (kDa)*	*No. of arms*	*Route*	*AUC (h·μg/mL)*	*t_1/2_ (h)*	*CL/F (mL/h/kg)*	*T_max_ (h)*	*C_max_ (μg/mL)*	*Vz/F (mL/kg)*	*Vss (mL/kg)*
sbC-PEGylated 17M-382	Y-shaped PEG+Y-shaped PEG	80 (40 + 40)	4 (2 + 2)	sc	2490 ± 67	107 ± 52.8	0.4 ± 0.1	48 ± 24	14.0 ± 3.4	59.7 ± 16.3	NA
				iv	1970 ± 240	72.0 ± 10.3	0.5 ± 0.1	NA	NA	NA	52.3 ± 12.5
PEGylated 17M-382	SUNBRIGHT GL2-400GS2	40	2	sc	449 ± 36	16.4 ± 0.9	2.2 ± 0.2	12.7 ± 9.9	16.3 ± 5.7	53.1 ± 7.2	NA
				iv	373 ± 30	17.4 ± 1.9	2.7 ± 0.2	NA	NA	NA	41.5 ± 11.0

Data are expressed as mean ± SD values (*n* = 3).

AUC, area under the curve; C_max_, maximum observed concentration; CL/F, clearance; NA, not applicable; t_1/2_, half-life; T_max_, time of maximum observed concentration; Vss, volume of distribution in the steady state; Vz/F, volume of distribution the terminal phase.

### The effects of sbC-PEGylation on the *in vitro* neutralizing activity of 17M-382

**FIG. 5.**
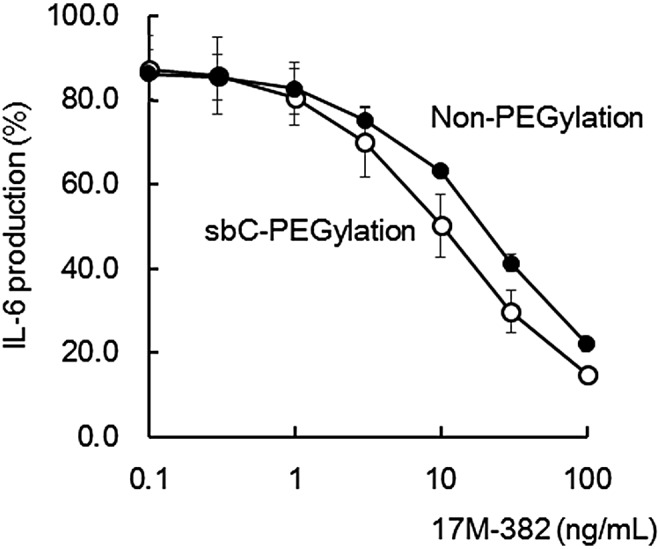
*In vitro* neutralizing activity of sbC-PEGylated aptamers. The IL-6 production induced by human IL-17A in NIH3T3 cells was inhibited by sbC-PEGylated 17M-382 (*open circles*) and non-PEGylated 17M-382 (*closed circles*). Data are expressed as the mean ± SD values of three independent experiments.

Since the PEGylation of pharmaceuticals sometimes reduces their biological activity [10], it is possible that sbC-PEGylation might decrease the neutralizing activity of the anti-IL-17A aptamers. Thus, we examined the effects of sbC-PEGylation on the neutralizing activity of 17M-382. Non-PEGylated 17M-382 inhibited the IL-6 production induced by IL-17A in a concentration-dependent manner (Fig. 5), and its IC_50_ value was 19.7 ± 6.3 ng/mL. On the other hand, the IC_50_ value of sbC-PEGylated 17M-382 was 10.0 ± 1.0 ng/mL. These results indicate that rather than reducing the *in vitro* neutralizing activity of the aptamers, sbC-PEGylation enhanced it by about twofold.

### The effects of sbC-PEGylation on the *in vivo* neutralizing activity of the aptamers

**FIG. 6.**
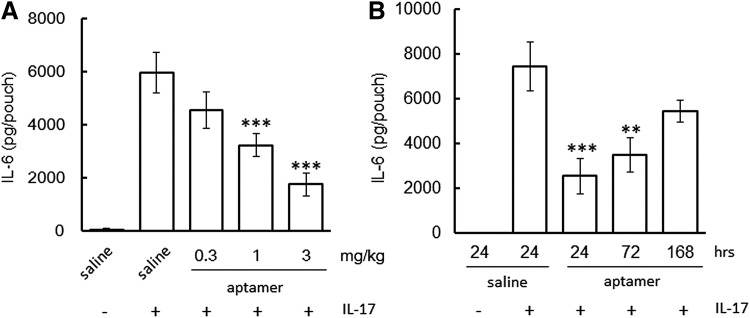
*In vivo* neutralizing activity of anti-IL-17A aptamers. The IL-6 production induced in air pouches by human IL-17A was inhibited by the intraperitoneal administration of sbC-PEGylated 17M-382. The aptamers were administered to the mice at 1 h **(A)** or 24, 72, or 168 h **(B)** before the administration of human IL-17A into the air pouches. Data are expressed as mean ± SEM values (*n* = 7 or 8). **P < 0.01, ***P < 0.001 compared with the saline+IL-17A-treated group (ANOVA followed by Dunnett's test). ANOVA, analysis of variance.

Next, we examined the effects of sbC-PEGylation on *in vivo* IL-17A-neutralizing activity by using a mouse air pouch model. In the saline-treated group, the IL-6 concentration of the air pouch was increased at 24 h after the administration of human IL-17A. However, the intraperitoneal administration of sbC-PEGylated 17M-382 1 h before the administration of IL-17A reduced the IL-6 concentration of the air pouch in a dose-dependent manner (Fig. 6A). Significant differences were observed between the IL-17A-neutralizing activity induced by 1 and 3 mg/kg of sbC-PEGylated 17M-382. Moreover, sbC-PEGylated 17M-382 continued to exhibit *in vivo* neutralizing activity for 72 h; that is, 3 mg/kg of sbC-PEGylated 17M-382 significantly reduced the IL-6 concentration of the air pouch (Fig. 6B). These findings indicate that sbC-PEGylated aptamers diffuse into inflamed tissues and that the *in vivo* neutralizing activity of sbC-PEGylated aptamers can be maintained for several days.

## Discussion

In this study, we reported a novel PEGylation method, sbC-PEGylation, which markedly improved the PK parameters and *in vitro* neutralizing activity of anti-IL-17A aptamers. Moreover, it was indicated that sbC-PEGylated aptamers diffuse into inflamed tissue and that they continue to exhibit *in vivo* neutralizing activity for 72 h after their administration. To the best of our knowledge, this is the first report to indicate that performing PEGylation with both symmetrical branching CED phosphoramidite and two-armed 40-kDa PEG improves the PK properties of aptamers, although Kang *et al.* reported that the conjugation of two linear PEG molecules and a symmetrical doubler prolonged the *in vitro* whole blood clotting time of an anti-thrombin DNA aptamer [20].

Figure 3 and Table 1 suggest that the MW of PEG (total: 80 kDa) and the steric hindrance induced by two 2-armed PEG inhibit the renal clearance and nuclease degradation of aptamers. Since the t_1/2_ of 170-kDa PEG in circulation was only 1.4 times longer than that of 50-kDa PEG [21], it was suggested that the conjugation of 80-kDa PEG might be an inefficient way of prolonging the plasma t_1/2_ of aptamers. However, the half-life of 17M-200-S1 that had been PEGylated with 80-kDa PEG was two times longer than that of 17M-200-S1 that had been PEGylated with 40-kDa PEG (Table 1). These results suggested that the use of two 40-kDa PEG might be responsible for improvement of PK properties by sbC-PEGylation. As for the number of arms, compared with two-armed PEG, there were no advantages of using four-armed PEG molecules when PEG was conjugated to aptamers by using conventional methods. On the other hand, the half-life of 17M-200-S1 that had been sbC-PEGylated with two 2-armed 40-kDa PEG molecules (total: four-armed 80-kDa PEG) was two times longer than that of 17M-200-S1 that had been PEGylated with four-armed 80-kDa PEG. These differences between sbC-PEGylation and PEGylation were not obvious when 17M-382, a stable oligonucleotide, was used. In addition, the plasma t_1/2_ of 17M-382 was markedly reduced when it was sbC-PEGylated with two linear 40-kDa PEG molecules. These results indicate that the 4 arms formed by sbC-PEGylation induce greater steric hindrance around oligonucleotides and prevent aptamers from being subjected to nuclease degradation.

PEGylation sometimes inhibits the biological activity of pharmaceuticals, although the loss of biological activity by PEGylation can be ameliorated by the associated increase in the molecule's half-life. It was reported that the PEGylation of an anti-VEGF aptamer reduced its *in vitro* biological activity by 25% [10]. On the other hand, the steric hindrance induced by sbC-PEGylation might have enhanced the binding of the anti-IL-17A aptamer to IL-17A, and hence, increased its *in vitro* neutralizing activity (Fig. 5). Ishiguro *et al.* also found that the PEGylation of Apt21-2 with a two-armed 40-kDa PEG molecule, SUNBRIGHT^®^ GL2-400GS2, increased its *in vitro* neutralizing activity against mouse IL-17A [17]. In addition, the *in vitro* neutralizing activity of anti-IL-17A aptamers against human IL-17A improved after their PEGylation with various kinds of PEG, including SUNBRIGHT GL2-400GS2, GL2-800GS2, and GL4-800GS2 (data not shown). Therefore, it was suggested that PEG might directly interact with IL-17A. Importantly, sbC-PEGylation was able to increase the *in vivo* pharmacological activity of anti-IL-17A aptamers by enhancing their neutralizing activity and prolonging their half-lives.

Figure 6A indicates that the sbC-PEGylated aptamers were able to easily diffuse into inflamed tissues after their administration. Aptamers are so small that they are able to recognize epitopes that antibodies cannot access [22]. In addition, it was reported that aptamers are able to efficiently penetrate into biological compartments [2]. Namely, their small size is one of the advantages that aptamers have over therapeutic antibodies. Based on PK studies, we used 80-kDa PEG (total MW of aptamer: 92 kDa) to prolong the half-life of aptamers *in vivo*. As a result, we found that the steric hindrance induced by sbC-PEGylation with two 2-armed 40-kDa PEG seemed to be greater than that associated with the use of 80-kDa PEG. In fact, the T_max_ (48 h) of sbC-PEGylated 17M-382 was 3.8 times longer than that (12.7 h) of PEGylated 17M-382 when they were administered to monkeys subcutaneously. However, since there were no differences in C_max_, Vz/F, or Vss between the sbC-PEGylated and PEGylated aptamers, it was suggested that sbC-PEGylated aptamers diffuse through the body in the same way as PEGylated aptamers. In particular, the sbC-PEGylated 17M-382 inhibited the production of IL-6 in air pouches when it was administered intraperitoneally 1 h before the injection of IL-17A (Fig. 6A). As for therapeutic antibodies, certolizumab pegol, a PEGylated Fab’ fragment of an anti-TNF-α antibody (total MW: 90 kDa), is able to penetrate inflammatory tissue more easily than other whole anti-TNF-α antibodies (total MW: 150 kDa, ref. 23). Taken together, it was suggested that the sbC-PEGylation of aptamers with 80-kDa PEG does not affect their ability to penetrate into inflammatory tissues.

sbC-PEGylation might be one way of overcoming the limitations associated with the modification of ribose and/or phosphate backbones after conventional SELEX, which are used to enhance nuclease resistance. Since these modifications sometimes reduce the pharmacological activity of aptamers, not all of the nucleotides of an aptamer can be chemically modified. In the case of anti-IL-17A aptamers, we synthesized >400 chemically modified aptamers and examined their *in vitro* neutralizing activity and stability in mouse serum. As a result, we identified 17M-382, in which 2 of its 31 nucleotides cannot be chemically modified. Disappointingly, the half-life of 17M-382 that had been conjugated to a conventional two-armed 40-kDa PEG molecule was only 17 h when it was administered to monkeys subcutaneously or intravenously. When it was compared with ARC15105, an anti-von Willebrand factor aptamer that has been PEGylated with 40-kDa PEG, which was selected by variant SELEX (t_1/2_: 67 h, iv; 65 h, sc; refs. 24 and 25), it was found that chemical modification could not completely prevent 17M-382 from undergoing nuclease degradation in monkeys. However, sbC-PEGylation markedly prolonged the half-life of 17M-382 (t_1/2_: 72 h, iv; 107 h, sc), which was consistent with the findings obtained for ARC15105.

IL-17A is a cytokine that is mainly produced by T helper 17 cells and plays a pathogenic role in systemic inflammatory diseases, such as multiple sclerosis, rheumatoid arthritis, psoriasis, etc. [26]. Thus, it is clear that anti-IL-17A aptamers have therapeutic potential as treatments for systemic inflammatory diseases. In fact, anti-IL-17A and anti-IL-17 receptor A aptamers inhibited the progression of experimental autoimmune encephalomyelitis (EAE), glucose-6-phosphate isomerase (GPI)-induced arthritis, collagen-induced arthritis, and osteoarthritis in mice [17,27,28]. It was also reported that the administration of anti-IL-17A antibodies ameliorated the symptoms of EAE, GPI-induced arthritis, and IL-23-induced dermatitis in mice [29–31], and clinical trials of anti-IL-17A antibodies, such as secukinumab and ixekizumab, revealed that the neutralization of IL-17A was successful as a therapy for psoriasis and ankylosing spondylitis (AS) in humans [32–34]. Since aptamers are considered to have some clinical advantages over therapeutic antibodies [1–3], anti-IL-17A aptamers might be an alternative to anti-IL-17A therapeutic antibodies. In particular, after its subcutaneous administration to monkeys, sbC-PEGylated 17M-382 exhibited a plasma t_1/2_ of 107 h, which was longer than the plasma t_1/2_ of albinterferon (human serum albumin-interferon α) in monkeys (91–92 h, ref. 35) and etanercept (a TNF receptor-Ig fusion protein) in humans (80 h, ref. 36). It is strongly suggested that sbC-PEGylation is a useful method for developing therapeutic anti-IL-17A aptamers for systemic inflammatory diseases, such as psoriasis and AS.

In conclusion, we report that sbC-PEGylation, a novel PEGylation method, improved the PK properties of anti-IL-17A aptamers. This study also suggests that the creation of new configurations using branching molecules (or doublers), spacers, and PEG could improve the PK properties of aptamers. In addition to modern oligonucleotide modification techniques, the use of novel PEGylation methods, such as sbC-PEGylation, could aid the development of therapeutic aptamers, especially for systemic disease.

## Supplementary Material

Supplemental data
