# Supplementary figures and images for: A Novel PEGylation Method for Improving the Pharmacokinetic Properties of Anti-Interleukin-17A RNA Aptamers
Source: Nucleic Acid Ther. 2017 Feb 1;27(1):36–44. doi: 10.1089/nat.2016.0627 (PMC5312557; doi:10.1089/nat.2016.0627)

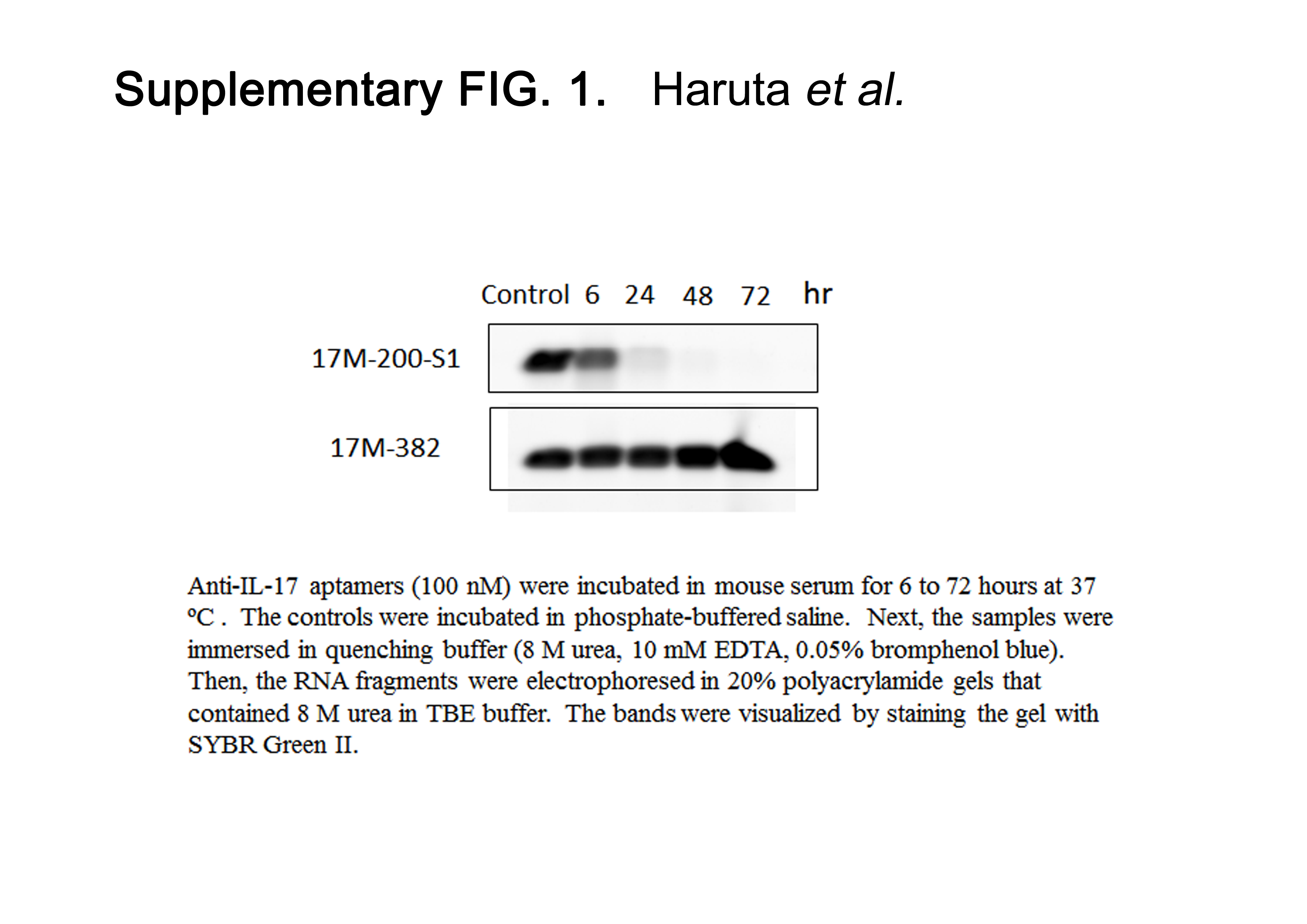

Supplement: Supplemental data [file Supp_Fig1.tif]
